# Induced Radionuclides and Their Activity Concentration in Gel Dosimeters Irradiated by Carbon Ion Beam

**DOI:** 10.3390/gels8040203

**Published:** 2022-03-23

**Authors:** Masumitsu Toyohara, Shinichi Minohara, Yohsuke Kusano, Hiroaki Gotoh, Yoichiro Tanaka, Masaru Yuhara, Yu Yamashita, Yoshiaki Shimono

**Affiliations:** 1 Research Initiative and Promotion Organization, Yokohama National University, 79-5 Tokiwadai, Hodogaya-ku, Yokohama 240-8501, Japan; tanaka-yoichiro-vw@ynu.ac.jp; 2 Toshiba Energy Systems & Solutions Corporation, Kawasaki 212-8585, Japan; masaru1.yuhara@toshiba.co.jp (M.Y.); yu.yamashita@toshiba.co.jp (Y.Y.); yoshiaki.shimono@toshiba.co.jp (Y.S.); 3 Kanagawa Cancer Center, Yokohama 241-8585, Japan; minoharas@kcch.jp (S.M.); y.kusano@kcch.jp (Y.K.); 4 Department of Chemistry and Life Science, Yokohama National University, Yokohama 240-8501, Japan; gotoh-hiroaki-yw@ynu.ac.jp

**Keywords:** micellar hydrogel, polymer hydrogel, carbon ion radiotherapy, radioactivity, Monte Carlo simulation

## Abstract

Radioactivity was measured in a micellar gel dosimeter, a polymer gel dosimeter, and water was irradiated by carbon ion beams at various beam energy conditions. Monte Carlo simulation was also performed to estimate the radioactivity. Short-lived positron-emitting nuclides were observed immediately after irradiation, but they decayed rapidly into the background. At 24 h post-irradiation, the dominant measured radioactivity was of ^7^Be. The simulation also showed minor activity of ^24^Na and ^3^H; however, they were not experimentally observed. The measured radioactivity was independent of the type of gel dosimeter under all irradiation conditions, suggesting that the radioactivity was induced by the interaction of carbon ions with water (the main component of the gel dosimeters). The ratio between the simulated and measured radioactivity was within 0.9–1.5. The activity concentration of ^7^Be was found to be less than 1/10 of the value derived using the exemption concept proposed by the International Atomic Energy Agency. This result should be applicable to irradiated gel dosimeters containing mainly water and 0–4 wt.% C and 0–1.7 wt.% N.

## 1. Introduction

Radiotherapy is widely used for treating cancer. Recently developed techniques, such as intensity-modulated radiotherapy, stereotactic radiosurgery, stereotactic radiotherapy, and stereotactic body radiotherapy, can deliver a high radiation dose to a three-dimensional (3D) tumor region with remarkably reduced side effects. Because these techniques typically create a high and constant dose in the tumor region and strong dose gradients around the boundaries, measurement of the 3D dose distribution is important. Furthermore, in order to maximize the potential of these techniques, operators should routinely verify the radiation delivery and monitoring systems, the patient positioning system, and treatment planning using the radiation beams. Due to these reasons safe, accurate, and convenient methods should be developed to measure the radiation intensity, radiation beam position, and dose distribution. Because gel dosimeters can easily measure the 3D dose with high accuracy, they have good prospects for meeting these needs in cancer treatment. Many types of gel dosimeters have been investigated [1,2,3,4,5]. For example, polymer gel dosimeters contain monomers that undergo polymerization induced by the radiation field, and the reaction depends on the absorbed radiation dose. Thus, one can measure the 3D dose distribution in terms of the polymerization degree, which may be characterized using magnetic resonance imaging (MRI) and optical computed tomography (OCT) methods [1,2,3,4,5,6,7]. Fricke gel dosimeters, which have been used in practice for more than half a century and undergone continuous improvements, can also measure the dose based on the conversion of ferrous ions (Fe^2+^) into ferric ions (Fe^3+^) in water. Under an irradiation field, various radicals are produced by water decomposition, which react with the surrounding species to convert Fe^2+^ into Fe^3+^. By measuring the distribution of Fe^3+^ using MRI or optical absorbance (OA), one can obtain the 3D dose distribution [1,2,3,4,8,9,10,11,12,13]. Although most gel dosimeters are not reusable, PVA-KI gel dosimeters are an exception. These gels are primarily composed of polyvinyl alcohol (PVA) and potassium iodide (KI) with a gelling agent. Since triiodide ions generated by radiation form a red-colored complex with PVA, one can measure the 3D dose distribution using the OCT method. Upon heating, the dosimeter returns to a colorless state under the reducing action of added fructose; thus, it could be reused [14,15,16,17]. Micellar gel dosimeters can also measure 3D dose distributions and thus have attracted considerable attention [18,19,20]. Because these dosimeters contain a small amount of Leucocrystal Violet dye, a coloration intensity distribution develops when the dye reacts with the radicals generated by radiation. Notably, the 3D dose distribution can be easily and quickly obtained by measuring this 3D coloration intensity distribution using the OCT method without requiring a special measurement environment [21].

Jordan et al. proposed a new type of micellar gel dosimeter with improved sensitivity [22]. We have also reported a novel micellar gel dosimeter [23]. Specifically, combining the gelator with a clay increased the sensitivity by about three times and reduced the scattering of X-ray radiation by about one fifth. Recently, we used this micellar gel dosimeter to measure the 3D dose distribution in carbon ion radiotherapy cancer treatments [24]. In this radiotherapy technique, a large amount of radiation energy is effectively focused on the tumor using a unique dose distribution: a low and constant dose near the surface of the body and a sharply distributed high dose at the end of the range, known as the Bragg peak. Because carbon ions have a high linear energy transfer (LET), their relative biological effectiveness (RBE) is greater than that of photon beams and proton beams (2–5 vs. 0.8–1.1) [25]. Therefore, carbon ion radiotherapy can remarkably reduce radiation exposure in patients [26,27]. Furthermore, because of the sharper penumbra of carbon beams [28], one can also minimize the energy deposition in normal tissues and/or important organs in the vicinity of the tumor [29]. Consequently, carbon ion radiotherapy can reduce the disease burden and improve the patient’s quality of life. To fully utilize the unique characteristics of carbon ion beams, it is important to accurately measure the 3D dose distribution. Although ionization chambers [30,31,32] and radiochromic films [32] have been used, they require longer measurement times compared to micellar gel dosimeters. Our research group has performed fundamental research on the generation of radicals in micellar gels irradiated by carbon ion beams [24], as well as a method for improving the coloration intensity distribution by referring to the reaction between the dye and radicals in another type of gel dosimeter [33].

A remaining challenge for the practical use of gel dosimeters is characterizing the radionuclides and their activity after they are irradiated by carbon ion beams. When a target is irradiated by a carbon ion beam, many types of lighter fragments are generated due to collisions with the primary carbon ions along the penetration path. Hartmann et al. and another group studied the dose of a carbon ion beam irradiation absorbed by water [34,35]. They denoted the fractions (residual number over the initial number) of primary carbon ions and lighter fragments as a function of water depth. Their results showed that the fraction of carbon ions gradually decreased from 1 to 0.5 and then decreased to 0 at the Bragg peak, while the lighter fragments (Z = 1–5, hydrogen–boron) gradually accumulated along the path. The fractions for Z = 1 and 2 reached their respective maximums of about 0.4 to 0.5 at the Bragg peak, and these values were notably one order of magnitude higher than those for Z = 3–5. Fragmentations with low energy also occurred under collisions between the carbon ions and the target nuclei, and these fragmentations further contained neutrons and certain radionuclides [29,36]. The neutrons generated the activation products in the gel material. The types of radionuclides and their activity depend on the primary beam, the gel composition, and irradiation conditions such as the beam energy, irradiation time, and cooling time. Taking cancer treatment for example, Gudowska et al. extensively studied the spatial distribution of the absorbed dose from the primary beam and the fragments including neutrons, based on experiments using a phantom and simulations [37,38,39]. Conversely, the residual radioactivity in gel dosimeters needs to be carefully considered for not only the application itself but also during their storage and disposal after use. However, there is limited information about the radioactivity of materials irradiated with carbon ion beams. A recent report from the International Atomic Energy Agency (IAEA) provided information on the selection and implementation of dismantling accelerators [40]. Although the report describes radionuclides commonly identified in spent solid materials and the procedure for disposing some spent devices, it does not mention specific disposal standard for devices irradiated by carbon ion beams. Rather, this report recommends measuring and calculating radioactivity in the devices before using them. In Japan, specific devices must be managed, such as radioactive wastes, if they are located in a medical accelerator with an accelerated energy above 2.5 MeV. For other irradiated devices, the authority also mandates the measurement and calculation of radioactivity to determine whether they should be considered radioactive waste. Nevertheless, knowledge is limited regarding the radionuclides and their activity in gel dosimeters irradiated with carbon ion beams. Yashima et al. performed extensive irradiation experiments using high-energy charged particles including carbon ions. They reported the cross sections and radionuclides in some metallic materials [41,42] but did not consider aqueous materials. Toshito et al. directly measured the fragmentation reaction induced in water by carbon ion beams using an innovative method and estimated the reaction’s cross section and radioactivity [43,44,45]. However, they did not report the induced radionuclides in water mixed with other materials (such as gel dosimeters). Soltani-Nabipour et al. estimated residual nuclides in water irradiated by carbon ion beams using the Monte Carlo code [46] without providing experimental confirmation.

As mentioned above, gel dosimeters may significantly improve various dose measurements in radiation cancer treatments and routine work related to the daily verification of radiotherapy systems. In addition, because water is the main component in both gel dosimeters and human soft tissue, gel dosimeters are useful for developing human-body-sized phantoms and for research in microdosimetry. Therefore, it is important to collect data for determining whether irradiated gel dosimeters should be treated as radioactive substances. To help manage spent gel dosimeters, this study aimed to clarify important radionuclides and their activity in spent gel dosimeters irradiated by carbon beams. The materials considered here are a micellar gel dosimeter and a polymer gel dosimeter. PVA-KI gel dosimeters and Fricke gel dosimeters are specifically excluded from this study because the heavy nuclei therein produce a wide range of products by spallation and nuclear fission [47]. We irradiated the two gel dosimeters and water under specific conditions directed by a therapy planning system. Then, we measured the gamma- and beta-emitting radionuclides as well as their activities. We also estimated the radioactivity by Monte Carlo simulation. The types of radionuclides, their activity, and the decay process were studied based on the experimental and simulation results. We used the obtained results to identify a radionuclide that is important for radioactive waste management. Then, the radioactivity concentration in the irradiated gel dosimeter was evaluated and compared to the value derived using the exemption concept proposed by the IAEA. Furthermore, we evaluated the applicability of Monte Carlo simulation to estimate the radionuclides and their activity by comparing the measured values with the calculated ones.

## 2. Materials and Methods

### 2.1. Specimens

Table 1a shows components of the micellar gel dosimeter and its elemental composition [9]. Because this gel contains > 90 wt.% water, we performed the same irradiation experiment on water for comparison. A polymer gel dosimeter (Table 1b) was also used. These dosimeters were prepared as according to previous studies [23,48]. The gel dosimeters and water were poured into separate cylindrical polyethylene containers (“U8” containers, with an inner diameter 48 mm, height 65 mm, wall thickness 1 mm; Sekiya Rika Co., Ltd., Tokyo, Japan) to a depth of 50 mm. The container has the same shape as the multi-nuclides standard volume source for gamma-emitting nuclides.

### 2.2. Carbon Ion Beam Irradiation

We irradiated the specimens with carbon ion beams at the Ion-beam Radiation Oncology Center in Kanagawa (i-ROCK). As of 2021, there were seven carbon ion radiotherapy facilities in clinical operation in Japan; i-ROCK started clinical operation at the Kanagawa Cancer Center in 2015. By using a synchrotron that accelerates carbon ions stepwise to 11 different energies (140, 170, 200, 230, 260, 290, 320, 350, 380, 400, and 430 MeV/u), beams generated at i-ROCK can create a complex 3D dose distribution in tumors. This is achieved using a range control combined fine-range shifter with multistep variable accelerated energy and 3D pencil beam fast scanning [49,50].

**Figure 1:**
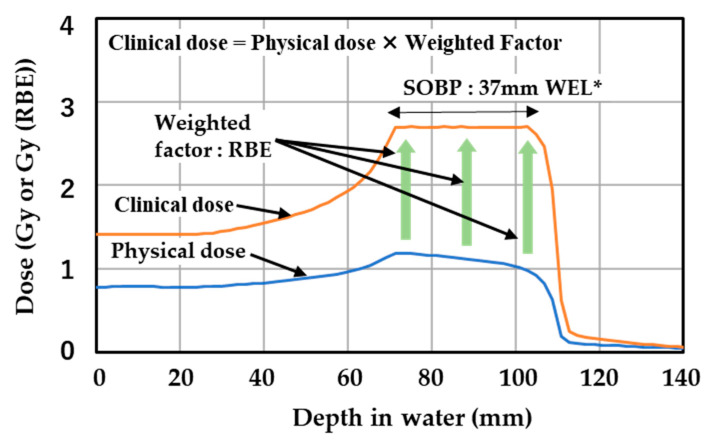
An example of clinical dose (Gy (RBE)), physical dose (Gy), weighted factor, and SOBP in carbon ion radiotherapy produced by the treatment planning system. The size and depth of the SOBP and the value of clinical dose are determined by the cancer type, the tumor size and location, the location of important organs, the size of margin for irradiation, and other conditions. WEL*: water equivalent length.

The irradiation condition was selected to produce a spread-out Bragg peak (SOBP) in the specimen by referring to the therapeutic method. Figure 1 describes the relationship between the clinical dose, physical dose, and SOBP in carbon ion radiotherapy. The clinical and physical doses are calculated by the treatment planning system. In Figure 1, the SOBP has a length of 37 mm and a clinical dose of 2.7 Gy (RBE) starting from a depth of about 70 mm. The uniform clinical dose distribution within the SOBP is produced by compensating for the variations in RBE and physical dose along the penetration depth. The physical dose distribution originates from superimposing multiple physical doses created by discretely changing the energy of the carbon ion beam. In the example, we introduce 23 carbon beams with set energies so that Bragg peaks were formed at intervals of 2 mm in water equivalent depth. The physical dose at each Bragg peak is adjusted by changing the number of irradiated carbon ions. The physical dose distribution becomes lower along the depth direction within the SOBP, due to variation in radiation quality of carbon ion beams along the penetration path inside the human body [51,52]. From the viewpoint of waste management, one must conservatively evaluate the radionuclides and their activity in spent gel dosimeters. Therefore, we set a constant physical dose within the SOBP, without using the condition that the physical dose decreases as the depth increases in radiation therapy.

**Figure 2:**
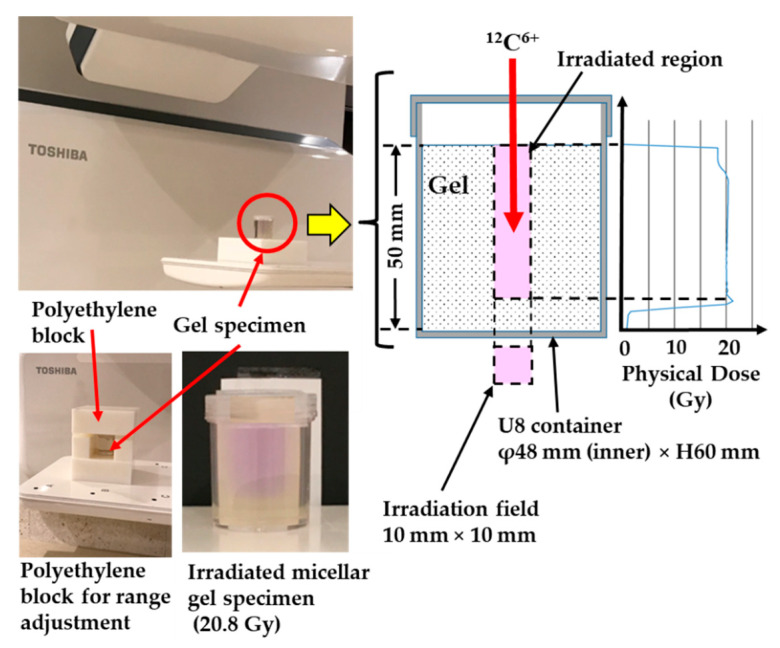
Setup of the gel specimen, irradiation geometry, and physical dose distribution.

The determined irradiation conditions for the specimens are shown in Figure 2. The figure illustrates the geometry and an example of the physical dose distribution produced by the therapy planning system. The irradiation region had the shape of a square with side lengths of 10 mm. A constant physical dose distribution to a depth of approximately 40 mm was produced using a superposition of 20 beams with different energies and different beam intensities. Table 2 lists the 10 experimental conditions selected for studying the radionuclides and their activity in the gel specimens and water. The beam conditions (A)–(C) are described in Table 3. In many cases, fractionated irradiation is performed to deliver the prescribed dose across multiple exposures. For example, patients with localized prostate cancer underwent carbon ion radiotherapy with 51.6 Gy (RBE) in 12 fractions over 4 weeks [53], and one fractionated dose was about 2 physical Gy. As shown in Table 1, water is the main component of the micelle gel dosimeter and the polymer gel dosimeter, which is similar to soft tissues including muscles in the human body. Therefore, we considered that carbon ion radiotherapy produces very similar physical doses in human organs (such as the prostate) and in these gel dosimeters. In order to conservatively evaluate the radioactivity, we irradiated the specimen to physical doses that are about 5, 10, and 15 times (10.4, 20.8, and 31.4 Gy, respectively) the aforementioned value used for prostate cancer. In experiment (7), irradiation was also performed with a mono- energy beam accelerated to 200 MeV/u. These physical doses were calculated by the treatment planning system. We monitored the dose by using the dose monitor at i-ROCK during the irradiation. In experiment (3), to homogeneously disperse the radionuclides, we dissolved the irradiated gel specimen by heating at 60 °C, which is above the melting point of gelatin. The effect of gamma-ray self-absorption in a bulk specimen on radioactivity measurement was estimated by comparison to other gel conditions, such as in experiments (1) and (2). Figure 2 shows that the irradiated region in the micellar gel specimen turned violet. When using a beam with high acceleration energy (beam conditions (B) and (C)), we adjusted the range of carbon ions to produce SOBP or Bragg peaks in the specimen by using a polyethylene block, as shown in Figure 2.

### 2.3. Measurement of Gamma-Emitting Radionuclides and Their Activity

We performed spectrometric measurements for gamma-emitting nuclides using a portable high-purity germanium-based radionuclide identifier (Falcon 5000, Mirion Technologies, Inc., Atlanta, GA, USA). The instrument has an energy range from 50 to 1500 keV, and its energy resolution (full width at half maximum (FWHM)) is not greater than 2.0 keV at 1332 keV and not greater than 1.0 keV at 122 keV. The number of channels is 4096. The spectra were acquired and analyzed using the GENIE-2000 Gamma Acquisition & Analysis software ver. 3.4 (Mirion Technologies, Inc.). The energy and peak efficiency were calibrated using a multi-nuclide standard volume source (Source Code MX033U8PP, Source Number 5123; source depth: 50 mm) supplied by the Japan Radioisotope Association. This standard volume source contained ^109^Cd, ^57^Co, ^139^Ce, ^51^Cr, ^85^Sr, ^137^Cs, ^54^Mn, ^88^Y, and ^60^Co of known activities.

### 2.4. Measurement of Beta-Emitting Nuclides and Their Activity

Before the irradiation experiments, we performed preliminary simulation and identified ^3^H as one of the dominant beta-emitting radionuclides 24 h after carbon ion beam irradiation. Consequently, we measured the activity of ^3^H using an automatic triple-to-double coincidence ratio liquid scintillation counter (300SL; HIDEX, Turku, Finland). The irradiated gel specimen was dissolved by heating at approximately 60 °C, followed by the distillation and collection of the condensation liquid. A liquid scintillation cocktail (13 mL, Insta-Gel Plus; PerkinElmer Co., Ltd., Waltham, MA, USA) and the condensation liquid (7 mL) were homogeneously mixed in a 20 mL vial tube, and ^3^H in the mixture was measured. The detection efficiency was calibrated using a ^3^H standard source (Water, [^3^H]; PerkinElmer Co., Ltd., lot number 1773956) with a specific activity of 0.037 GBq/g.

### 2.5. Simulation Configuration

Because there is only limited knowledge of radionuclides formed in gel specimens irradiated by carbon ion beams, we theoretically evaluated the radionuclides and their activities using PHITS (version 3.24) and DCHAIN-SP2014 codes. PHITS is a 3D multipurpose code for particle and heavy ion Monte Carlo transport [54], and DCHAIN is a code for calculating high energy particle-induced radioactivity [55]. The input parameters for DCHAIN were automatically generated using the PHITS tally (T-Dchain). The irradiation system was set in reference to the irradiation equipment layout of the HIMAC of the National Institute of Radiological Sciences [50]. Table 4 shows the elemental compositions of the specimens used for the simulation.

We introduced a simple geometry to reveal the induced radionuclides and their decay processes after irradiation. Carbon ion beams with projectile energies of 140, 200, or 400 MeV/u were irradiated along the axial direction of the cylindrical gel specimen (diameter: 48 mm, height: 50 mm). In the case of 140 MeV/u irradiation, the Bragg peak occurred at a penetration depth of approximately 40 mm from the specimen surface. For other projectile energies, we adjusted the range of carbon ions to form a Bragg peak at the same depth by setting a polyethylene block in the travelling path of the carbon ions.

**Figure 3:**
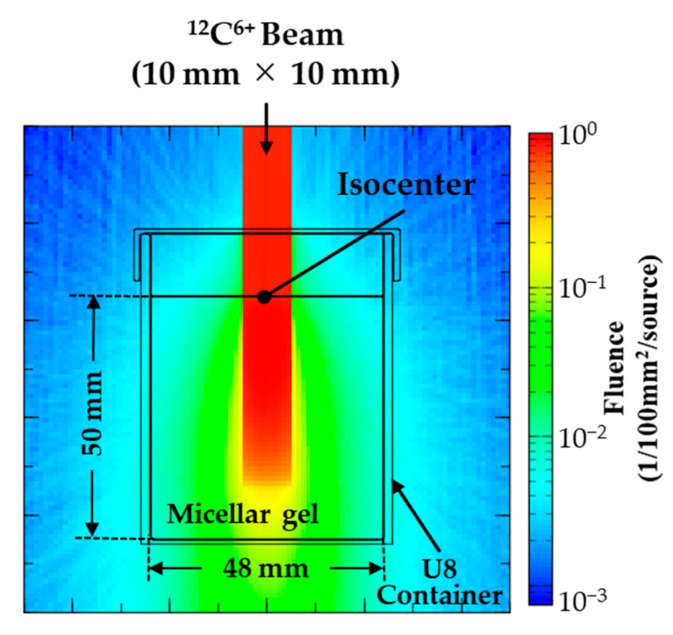
Irradiation of a micellar gel specimen with carbon ion beam under beam condition (A) and the resulting fluence.

When simulating therapeutic scenarios, the irradiation conditions (such as the projectile energies and number of carbon ions, and the shapes of gel specimen and polyethylene block used for range adjustment) were set to match those used in the experiments. Figure 3 displays representative simulation results obtained using all the 20 beams of condition (A) specified in Table 3. We found that the particle fluence was large in the irradiation area and small outside it. Furthermore, the particle fluence was constant at approximately 1.0 (1/100 mm^2^/source) from the upper surface to a depth of approximately 35 mm in the gel specimen. Below this region, it rapidly decreased and reached approximately 0.1 (1/100 mm^2^/source) at the bottom of the container. The total number of the Monte Carlo sampled particle histories was set to 100,000. The maximum relative statistical error was 0.7% in the SOBP region (10 mm × 10 mm × 40 mm (depth)).

**Figure 4:**
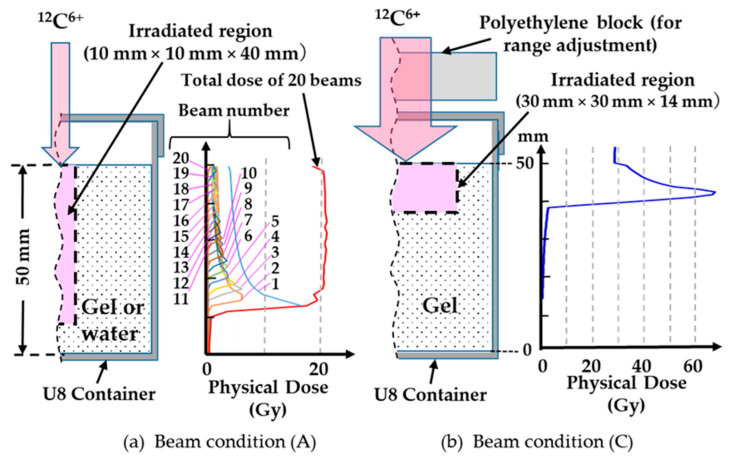
Irradiation geometries under beam conditions (A) and (C) and the corresponding dose distributions. (**a**): experiment (4), (**b**): experiment (7).

Figure 4 presents the physical dose distributions under experimental conditions (4) and (7). SOBP was verified in the specimen under a superposition of 20 beams (beam condition (A)), and a single Bragg peak was produced at a mono-energy of 200 MeV/u (beam condition (C), 1 beam). The total number of the Monte Carlo sampled particle histories was set to 500,000, and the maximum relative statistical error was 1.6% in the irradiated region.

## 3. Results and Discussion

### 3.1. Radionuclides Identified a Short Time after Irradiation

**Figure 5:**
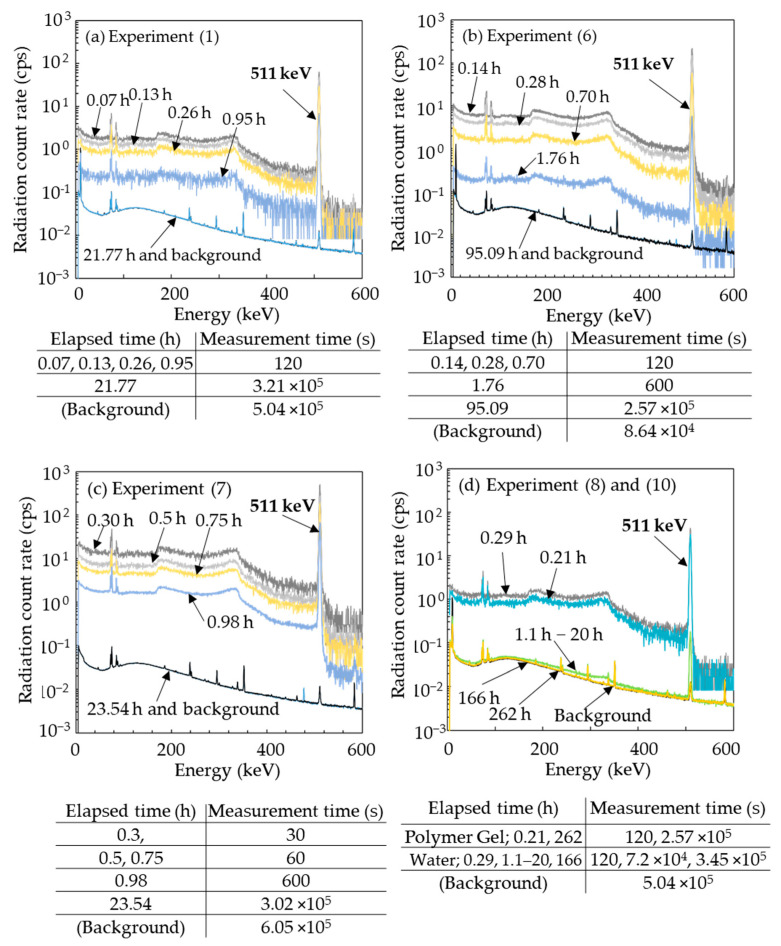
Gamma-ray spectra of selected specimens at different times after irradiation with carbon ion beam. (**a**): experiment (1), (**b**): experiment (6), (**c**): experiment (7), (**d**): experiment (8) and (10).

Figure 5 shows the typical gamma-ray spectra detected from the gel specimen a short time post irradiation under specific beam conditions. Figure 5a–d show the results for experiments (1), (6), (7), and (8 and 10). In all cases, there was a strong photopeak at 511 keV and Compton scattering immediately after irradiation. The radiation count rates decreased rapidly over time. Changes in the spectral shape during decay were also very similar in all experiments. The spectrum of the irradiated specimen was indistinguishable from the background after a certain period of time, which was approximately 24 h in experiments (1) and (2) and much longer in experiments (6), (8), and (10). Experiments (6) and (7), which were performed at a higher dose, showed higher count rates immediately after irradiation than experiments (1), (8), and (10).

**Figure 6:**
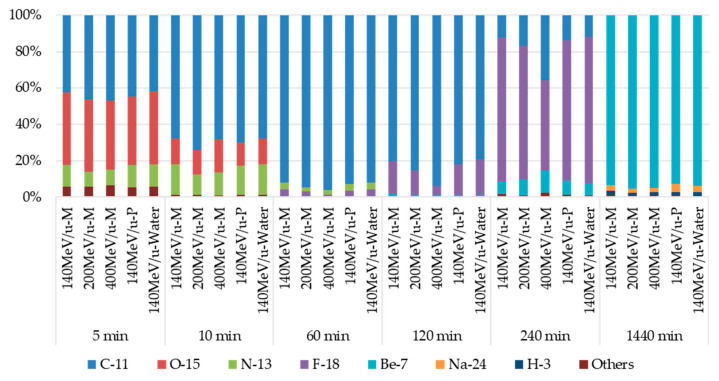
Simulation results of induced radionuclides and the ratio of their activities from 5 min to 24 h (1440 min) after irradiation. M and P denote a micellar gel specimen and a polymer gel specimen, respectively.

Some previous Monte Carlo simulations showed the production of fragments, including positron-emitting radionuclides such as ^15^O and ^11^C in water as the target material, due to spallation immediately after irradiation [46,56]. These are short-lived nuclides that emit a positron, which in turn combines with a nearby electron to produce two 511 keV photons during annihilation. Consequently, we also evaluated the radionuclides and their decay processes from simulations under the simple geometry described in Section 2.5. Figure 6 shows the induced radionuclides and the ratio of their activities within 24 h after irradiation. The types of radionuclides and their activity ratios were approximately the same regardless of the beam energy and the specimen type. Approximately 5 min after irradiation, the dominant nuclides were ^15^O (half-life 122.24 s) [57], ^11^C (half-life 20.39 min) [57], and ^13^N (half-life 9.965 min) [57]. Between 10 and 120 min, the dominant nuclide was ^11^C, and approximately 240 min after irradiation ^18^F (half-life 109.77 min) was dominant [57], as shown in Figure 6. Note that these are all positron-emitting radionuclides that generate two 511 keV photons, and their total activities exceeded approximately 90%. After 240 min, the dominant radionuclide was ^7^Be (half-life 53.12 days, photon energy 477.959 keV) [57], and its activity was approximately 95% of the total at 24 h after irradiation. The other nuclides from the simulation were ^24^Na (half-life 14.959 h) [57] and ^3^H (half-life 12.3 years) [57], but their activity was only 3.0% and 1.5% of the total activity, respectively.

**Figure 7:**
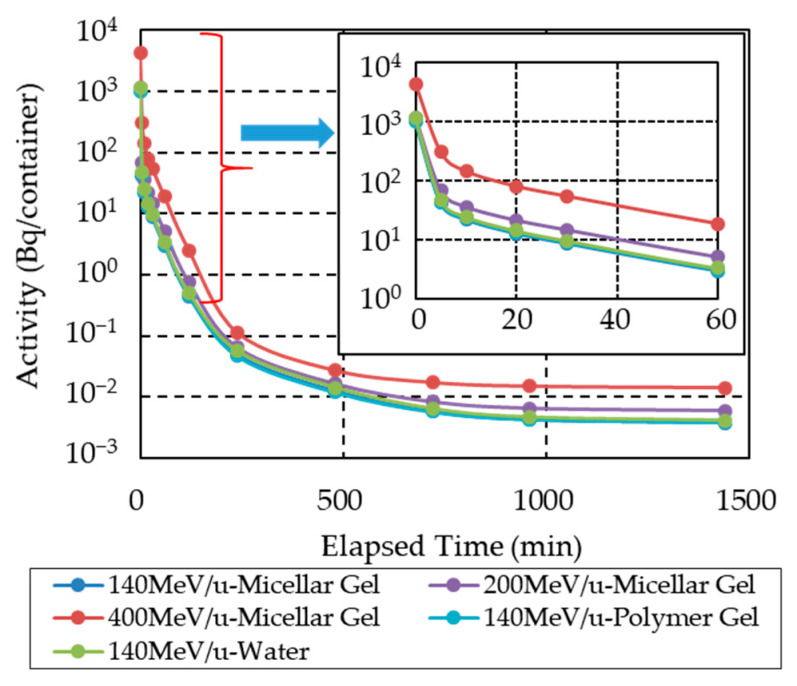
Simulated decay of total radioactivity under selected irradiation conditions. The simulations were carried out in the simple geometry configuration.

Figure 7 shows the simulated radioactivity decay for selected specimens. The calculation was performed under the simple geometry configuration (see Section 2.5). The same physical dose was deposited in each sample. We found that the decay of radioactivity was similar for all sample types and irradiation conditions. As shown in experiments (1), (7), (8), and (10) (c.f. Figure 5), the radiation count rates also decreased similarly regardless of the specimen type and irradiation conditions, reaching the same value after about 24 h. The calculated results shown in Figure 6 agree with the experimental ones. Furthermore, our simulation results indicated that the radioactivity depended on the beam energy.

Hence, we conclude that the short-lived radionuclides, ^13^N, ^15^O, ^11^C, and ^18^F, were generated in the specimens soon after carbon ion beam irradiation, and their decay produced 511 keV photons that were observed as photopeaks at the same energy as shown in Figure 5. After the rapid decay of these radionuclides, ^7^Be became the dominant radionuclide approximately 240 min after irradiation. There were no differences between the gel and water specimens. The radioactivity depended on the physical dose and the beam energy for the superposition of carbon ion beams.

### 3.2. Radionuclides Identified Approximately 24 h after Irradiation

**Figure 8:**
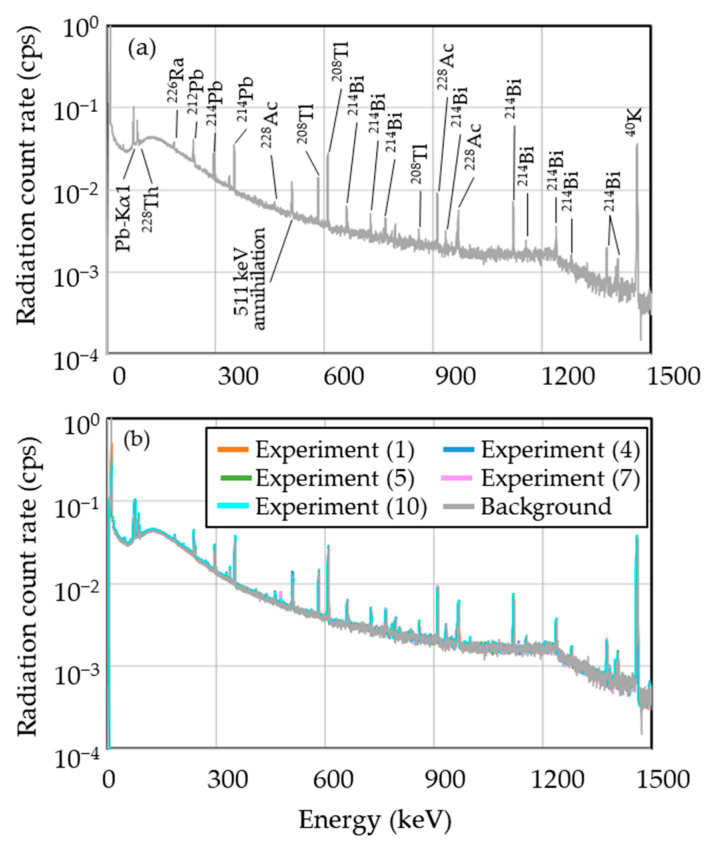
Gamma-ray spectra of (**a**) the background and (**b**) selected specimens measured about 24 h after irradiation.

Figure 8 shows all measured gamma-ray spectra for the gel specimens and water approximately 24 h after irradiation. The background spectrum is also shown. We found no differences between the spectra of all specimens under different irradiation conditions or between the irradiated specimens and background. Based on the aforementioned simulation results, we hypothesized that ^7^Be is the dominant radionuclide and ^24^Na and ^3^H are the minor radionuclides in the gel specimen.

### 3.3. Identification of ^7^Be

**Figure 9:**
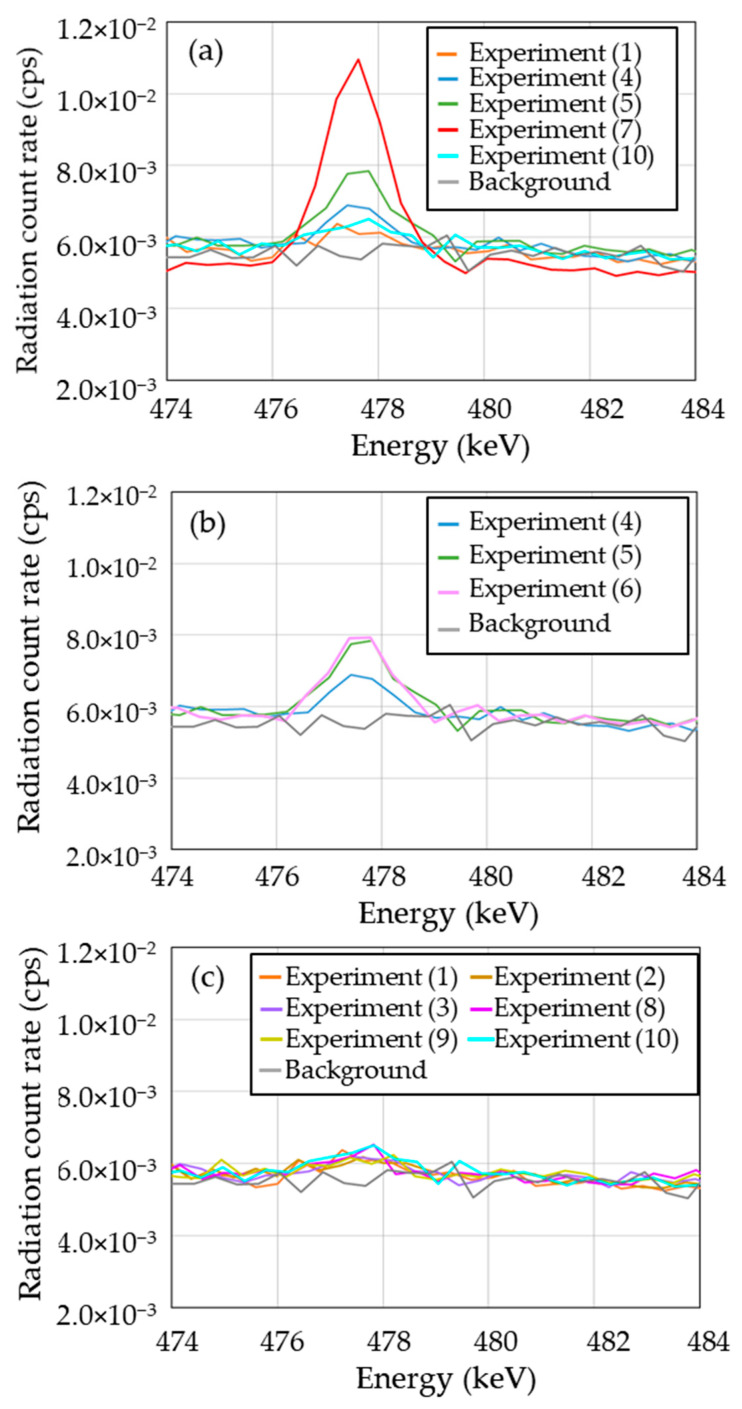
Energy spectra of all irradiated specimens and background in the range of 474–484 keV. (**a**): effect of physical dose, (**b**): effect of beam energy, (**c**): effect of specimen type.

Tilley et al. [58] and Doering [59] showed that ^7^Be (half-life 53.3 days) decays by capturing an electron to form ^7^Li in either the ground state or the first excited state, with a branching ratio of 10.44% to the excited state. The decay to ground state ^7^Li is accompanied by the emission of gamma rays at an energy of 477.959 keV [58,59]. Hence, we measured the activity of ^7^Be using gamma-ray emission. Figure 9 shows the energy spectra for all irradiated specimens and the background in the range of 474–484 keV. Clear photopeaks were observed in some cases. In order to confirm the identification of ^7^Be, we re-calibrated the energy using the photopeaks of natural radionuclides ^214^Pb (photon energy: 351.923 keV) [57] and ^241^Bi (photon energy: 609.312 keV) [57]. For the same purpose, the decay of radiation count rate for a clear photopeak in the energy range between 476 and 479 keV was measured. From these results, we concluded that these photopeaks originated from ^7^Be. As shown in Figure 9a, clear photopeaks occurred in experiments (4) and (5), which used the respective physical doses of 20.8 and 31.2 Gy to produce the SOBP beam conditions. Furthermore, a large photopeak occurred in experiment (7), using the 200 MeV/u mono-energy beam condition. The radiation count rate depends on the physical dose. At the same dose, the radiation count rate for the superposition of high-energy beams (beam condition (B)) is higher than that of low-energy beams (beam condition (A)), as shown in Figure 9b. On the other hand, as observed in Figure 9c, there is no clear photopeak at the physical dose of 10.4 Gy. There were also no apparent differences in the energy spectra between the gel specimens and water. The radiation count rates were slightly higher for the gel and water specimens than that for the background. These results confirmed the generation of ^7^Be in the irradiated specimens. Table 5 shows the measured activity of ^7^Be in all specimens. This activity was 5 ± 5 × 10^−1^ Bq/specimen in both experiments (4) and (5). In the case of 200 MeV/u mono-energy irradiation (experiment (7)), the activity was 14 ± 5 × 10^−1^ Bq/specimen. Although a clear difference was not observed in the spectra for experiments (1), (2), and (3), ^7^Be was only detected in experiment (3), and the activity was 2 ± 4 × 10^−1^ Bq/specimen. These results were regarded as the effect of gamma-ray self-absorption, although its impact on ^7^Be measurement was small. We could not detect radioactivity from the other micellar gels, the polymer gel specimens (experiments (8) and (9)), or water (experiment (10)), as shown in Table 5. The detection limit was set to three times the counting error (3σ counting error, [60]) of ^7^Be in each specimen, and its value was 2 Bq/specimen for all experiments except for experiment (2) (3 Bq/specimen).

**Figure 10:**
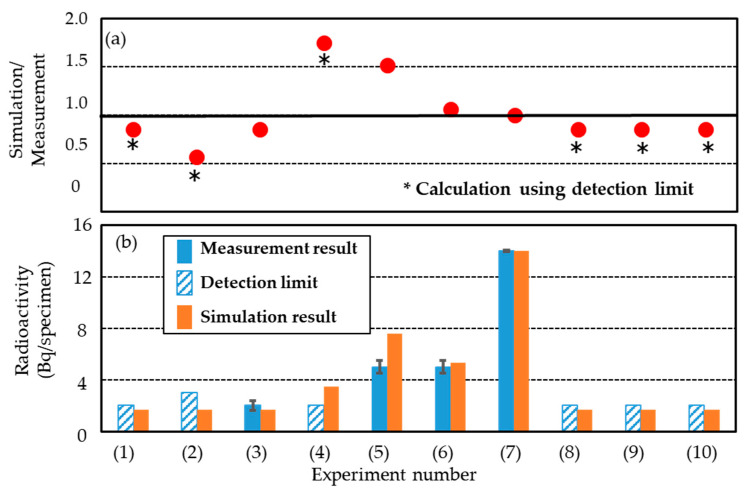
Comparison of measured and simulated ^7^Be radioactivities. (**a**) Ratio of simulated radioactivity to the measured value. (**b**) Measured and simulated radioactivities. The 1σ counting error intervals are shown as error bars in the measurement results.

Figure 10 compares the measured and simulated activities of ^7^Be. The measured results for experiments (1), (2), (4), (8), (9), and (10) are close to the detection limits in Figure 10b. In the cases where ^7^Be was detected, there was good agreement between the measured and simulated results. The ratio of simulated to measured values ranged from 0.9 to 1.5. The simulated result was generally higher (except for experiment (3)), and the agreement was the worst in experiment (4). In particular, this ratio was close to 1 for experiments (6) and (7). These results led us to believe that the ^7^Be activity in irradiated gel dosimeters can be either accurately or conservatively evaluated using PHITS and DCHAIN codes by assuming the exact irradiation geometry and experimental conditions.

### 3.4. Identification of ^24^Na and ^3^H

^24^Na decays to the stable ^24^Mg by emitting an electron and two main gamma-ray photons (1368.633 and 2754.028 keV), with half-lives of 14.959 h [57]. Based on this information, we examined the gamma-ray spectra measured at an energy of approximately 1368 keV for all specimens but could not observe any photopeaks of ^24^Na. The detection limit was 0.6 Bq/U8 specimen (3σ counting error) for all experiments. According to the simulation results in Figure 6, the activity of ^24^Na in the specimen was estimated to be approximately 1/30 times that of ^7^Be. We think that the absence of ^24^Na photopeaks is due to its short half-life as well as its small amount generated in the irradiated specimen.

^3^H undergoes beta decay to produce ^3^He and releases an energy of 18.6 keV, with a half-life of 12.32 years [57]. Because our simulation predicted a very small amount of generated ^3^H, we only measured the specimen in experiment (7) subjected to the highest irradiation. Still, we could not detect ^3^H (detection limit: 0.1 Bq/specimen, 3σ counting error). Because simulation for this experiment yielded a ^3^H activity of about 0.2 Bq/U8 specimen (U8 container), we think that its activity is near the detection limit of the liquid scintillation counter.

### 3.5. Effect on Induced Radioactivity

**Figure 11:**
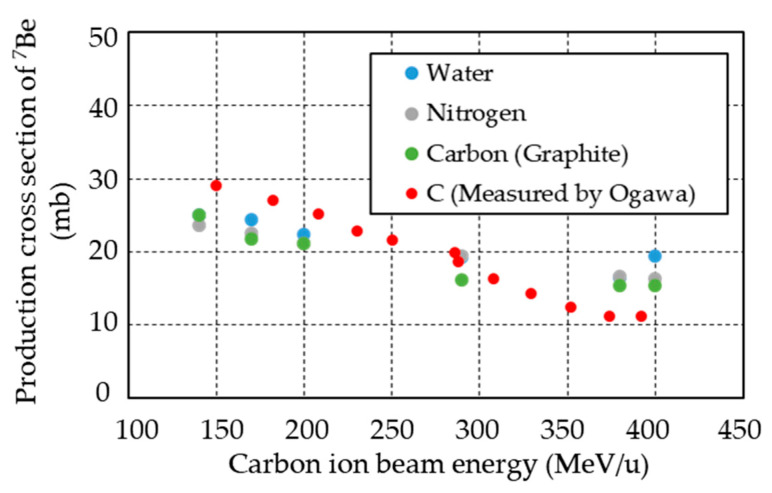
Production cross section of ^7^Be in water, carbon, and nitrogen as target, calculated using “PHITS” code and experimentally measured by Ogawa et al. [61].

From the aforementioned results, we concluded that the type of radionuclides and their relative activity ratio in irradiated specimens were independent on the type of gel dosimeter (micellar vs. polymer). This was probably due to the fact that water was the main constituent in both gel dosimeters according to Table 1a,b, despite small amounts of carbon and nitrogen. Ogawa et al. reported the production cross section of ^7^Be by measuring the fragmentation cross section of the ^12^C(^Nat^C,x)X reaction [61]. They irradiated natural carbon (graphite) as a target with carbon ion beams at an energy of 100 to 400 MeV/u and reported ^7^Be as one of the main generated nuclides (production cross section: 11 to 30 mb). Figure 11 shows the production cross section of ^7^Be for water, carbon, and nitrogen targets calculated by “PHITS”, as well as the measurement results by Ogawa et al. From the figure, the production cross section of ^7^Be is the same regardless of the type of targets, indicating that ^7^Be is mainly produced by the spallation of carbon ions. More importantly, the dominant radionuclide in the micellar and polymer gel dosimeters in this study (containing approximately 4 wt.% carbon and approximately 1.7 wt.% nitrogen) can be regarded as ^7^Be. We considered the other minor components to have little effect on the generation of radionuclides under carbon ion irradiation. From these considerations, we concluded that the radioactivity of ^7^Be does not depend on the composition of the gel dosimeter, at least when the carbon and nitrogen contents are within 0–4 wt.% and 0–1.7 wt.%, respectively.

On the other hand, the radioactivity depended on not only the physical dose but also the beam energy. The latter dependence can be explained as follows. Geithner et al. showed that the energy loss of carbon ions in water is a function of their energy [62]. When the carbon ions have a higher energy, the position of the Bragg peak moves deeper into the specimen while its height decreases, owing to energy loss along the penetration path. In experiments (4) and (6), we produced a constant physical dose distribution in the specimen using a superposition of 20 beams with different energies and different beam intensities. To clarify the difference in beam energy between these two experiments, we present the “averaged beam energies” in Table 2 and Table 5. The “averaged beam energy” was calculated by weighting 20 beam energies with their intensities (for the calculation method see the footnote of Table 2). The averaged beam energy was 120 MeV/u in experiment (4) and 389 MeV/u in experiment (6). The fluence of ^7^B in the specimen in these cases was 1.3 × 10^−4^ and 7.8 × 10^−4^ (1/100 mm^2^/source) in experiment (4) and experiment (6), respectively, with the latter about 6 times larger than the former. This means that high-energy carbon ions travel a longer distance in the medium and produce greater fragmentation. As a result, it was considered that the radioactivity of ^7^Be in experiment (6) was higher than that in experiment (4).

### 3.6. Consideration of Radioactive Waste in Spent Gel Dosimeters

Our simulation and experimental results indicated strong radiation from the gel dosimeters immediately after irradiation due to positron-emitting radionuclides with short half-lives. Later, the dominant radionuclide was ^7^Be at 24 h after irradiation, and the other nuclides were minor. Therefore, we believe that it is unnecessary to consider the influence of strong gamma rays at least after 24 h when managing the irradiated material. Our results confirmed that the radioactivity concentration of ^7^Be is important. In addition, we found that the radioactivity concentration is similar between the micellar gel dosimeter and polymer gel dosimeter tested here. The following discussion focuses on the radioactivity concentration of ^7^Be was considered from the viewpoint of radioactive waste management.

Different countries have different standards regarding when irradiated materials should be considered radioactive wastes. However, such standards do not exist for gel dosimeters, which are still under development and not used in actual radiotherapy. Therefore, we referred to the activity concentrations for bulk amounts of material containing radionuclides of artificial origin, which was derived using the exemption concept proposed by the IAEA [63]. To be exact, one could not compare the activity concentration of ^7^Be proposed by the IAEA with that of the irradiated gel dosimeter itself. However, it is important for researchers to know the activity concentration when applying gel dosimeters to measure the 3D dose distribution in carbon ion radiotherapy. The measured activity concentrations of ^7^Be and their value proposed by the IAEA are listed in Table 5. For a conservative estimation, we assumed that all the ^7^Be was concentrated in the irradiated region of the specimen. When ^7^Be could not be detected, we used the activity of the detection limit. Our measured activity concentration of ^7^Be was less than 1/10 of the value proposed by the IAEA. For reference, we also listed the activity concentration of ^3^H in Table 5, where the specimen was subjected to irradiation 20 times higher than that used in practical carbon ion radiotherapy. From the conservative experimental results and conservative estimation of the activity concentration, we believe that the ^7^Be generated in gel dosimeters irradiated by carbon ions under therapeutic conditions is not an important issue for nuclear safety.

## 4. Conclusions

In order to develop gel dosimeters for clinical applications, their radioactivity following carbon ion irradiation was estimated for the first time. Specifically, we irradiated a micellar gel, a polymer gel, and water (the gels’ main component) with carbon ion beams and measured the induced radionuclides and their activities at different times after irradiation. For a conservative estimation, the dose deposited in the specimens was 5–15 times of that used in practical carbon ion radiotherapy. We also calculated the radioactivity using Monte Carlo simulation code PHITS and the related code DCHAIN to help analyze the experimental results. Immediately after irradiation, there was strong radiation due to short-lived positron-emitting radionuclides such as ^11^C, ^16^O, ^13^N, and ^18^F. After 24 h, the aforementioned nuclides were no longer observed due to their rapid decay, and the only measured radionuclide was ^7^Be. These experimental observations were supported by our simulation, which also showed the generation of positron-emitting radionuclides. After 24 h, those nuclides were no longer observed due to their rapid decay, and the only measured radionuclide was ^7^Be. These experimental observations were supported by our simulation, which also showed the generation of positron-emitting radionuclides and ^7^Be becoming the dominant nuclide after 24 h with minor amounts of ^24^Na and ^3^H (whose activities were less than 1/30 that of ^7^Be). Moreover, the simulation revealed that the two gel dosimeters and water produced similar types of radionuclides with comparable relative activity ratios under all irradiation conditions. Because water was the main component in both gels, most of the induced radionuclides were generated from the interaction of carbon ions with water. Additionally, our measurement and simulation both showed that the activity is higher when using a higher beam energy, due to the more numerous fragments produced by the interaction of carbon ions with the specimen material over a longer distance. The ratio of the simulated radioactivity to the measured one falls within the range of 0.9–1.5, indicating the feasibility of using Monte Carlo simulation to estimate the radioactivity in specimens irradiated by carbon ions. These results led us to conclude that short-lived positron-emitting radionuclides are not a problem for radiation management at least at 24 h or longer after irradiation, and that the dominant source of radioactivity in irradiated gel dosimeters is ^7^Be. Under practical irradiation conditions that form SOBP in carbon ion radiotherapy, ^7^Be produced in gel dosimeters irradiated at even a high physical dose of 31.8 Gy had a radioactivity of 5 ± 5 × 10^−1^ Bq/specimen. In the case of mono-energy irradiation, the irradiated gel dosimeter contained 14 ± 5 × 10^−1^ Bq/specimen at a physical dose of 69 Gy at the Bragg peak. We also conservatively estimated the activity concentrations of ^7^Be in these samples, and found the values to be less than 1/10 of that derived using the exemption concept proposed by the IAEA. The most important findings in this study are the identification of ^7^Be as the dominant radionuclide and the estimation of its radioactive concentration in irradiated gel dosimeters with specific compositions (mainly water and 0–4 wt.% C and 0–1.7 wt.% N) against the IAEA value by considering the production cross section of ^7^Be.

## Figures and Tables

**Table 1:** Composition of the two gel dosimeters.

(**a**) Micellar gel dosimeter *		
**Component**	**Weight percent (%)**	**Supplier/Grade**
**Water**	91.6	Ion exchange water
**Gelatin**	4.95	Nacalai Tesque, Inc. 16631-05
**“TritonX-100”**	2.86	Alfa Aesar, 9002-93-1
**CH_2_Cl_2_**	4.06 × 10^−1^	Wako Co. Ltd., 044-28305
**Nano-clay**	1.53 × 10^−1^	BYK Japan KK
**LCV**	6.60 × 10^−3^	Wako Co. Ltd., 321-56072
(**b**) Polymer gel (PAGAT Gel) dosimeter **		
**Component**	**Weight percent (%)**	**Supplier/Grade**
**Water**	89	Ion exchange water
**Gelatin**	5.0	Sigma Aldrich G2500-500G, PREMIUM
**Acrylamide**	3.0	TCI A0139
***N*** **,*N*-Methylene Bisacrylamide**	3.0	Sigma Aldrich 146072-500G
**THPC**	1.3 × 10^−2^	TCI T0607

* Specific density: 1.005 g/cm^3^; “TritonX-100”: surfactant; LCV: Leucocrystal Violet, a cationic triarylmethane dye. ** specific density: 1.0 g/cm^3^; PAGAT: poly acrylamide, gelatin, and THPC; THPC: Tetrakis (hydroxymethyl) phosphonium chloride.

**Table 2:** Experimental conditions.

No.	Specimen	Physical Dose	Beam Condition	Averaged Beam Energy	Remark
(1)	Micellar gel	10.4 Gy at SOBP	(A)	120 MeV/u *	
(2)		10.4 Gy at SOBP			
(3)		10.4 Gy at SOBP			Dissolve by heating after irradiation
(4)		20.8 Gy at SOBP			
(5)		31.2 Gy at SOBP			
(6)		20.8 Gy at SOBP	(B)	389 MeV/u *	
(7)		69 Gy at Bragg peak	(C)	200 MeV/u	Mono- energy
(8)	Polymer gel	10.4 Gy at SOBP	(A)	120 MeV/u *	
(9)		10.4 Gy at SOBP			
(10)	Water	10.4 Gy at SOBP			

* Averaged beam energy = Σ *Ii ×* E*_eff_*_,i_ for i = 1–20; *Ii*: beam intensity ratio of the *i*-th beam, under the constraint Σ *Ii* = 1.0 for *i* = 1–20; E*_eff,i_*: beam energy of the *i*-th beam shifted by the range shifter (RSF).

**Table 3:** Beam parameters used for beam conditions (A)–(C).

Beam Number	Beam Condition (A)		Beam Condition (B)		Beam Condition (C)	
	Energy (MeV/u)	RSF (mm WEL) *	Energy (MeV/u)	RSF (mm WEL) *	Energy (MeV/u)	RSF (mm WEL) *
1	170	18.1	400	0	200	0
2	140	1.16	400	2.09	—	
3	140	3.25	400	3.94		
4	140	5.10	400	6.03		
5	140	7.19	400	7.88		
6	140	9.04	400	9.97		
7	140	11.13	400	12.06		
8	140	13.22	400	15.15		
9	140	15.08	400	16.01		
10	140	17.17	400	18.10		
11	140	19.02	400	19.95		
12	140	21.11	400	22.04		
13	140	22.96	380	2.09		
14	140	25.29	380	3.94		
15	140	27.14	380	6.03		
16	140	29.23	380	7.88		
17	140	31.09	380	9.97		
18	140	33.18	380	11.82		
19	140	35.03	380	13.92		
20	140	37.12	380	16.01		

* Water equivalent depth (WED) for the range shifter (RSF).

**Table 4:** Elemental composition (%) of the two gel dosimeters and water used in simulation.

Specimen	H	Li	C	N	O	Na	Mg	Si	P	S	Cl
**Micellar gel**	10.9	4.00 × 10^−4^	3.98	7.49 × 10^−1^	84.0	4.60 × 10^−3^	2.65 × 10^−2^	4.50 × 10^−2^	-	5.80 × 10^−3^	3.39 × 10^−1^
**Polymer gel**	12.3	-	3.50	1.68	82.5	-	-	-	3.13 × 10^−2^	5.51 × 10^−3^	3.54 × 10^−2^
**Water**	11.1	-	-	-	88.9	-	-	-	-	-	-

**Table 5:** Measured radioactivity in all the experiments after irradiation by carbon ion beams.

Experiment	Specimen	Physical Dose (Gy)	Averaged Beam Energy * (MeV/u)	Radio-nuclide **	Bq/specimen	Radioactivity Concentration (Bq/g) ***	Remark
(1)	Micellar gel	10.4	120 *	^7^Be	N.D. (<2)	< 5 × 10^−1^	IAEA Safety Guide No. RS-G-1.7 [39] ^7^Be; 10 Bq/g ^3^H; 100 Bq/g
(2)		10.4		^7^Be	N.D. (<3)	< 1	
(3)		10.4		^7^Be	2 ± 4 × 10^−1^	5 × 10^−1^	
(4)		20.8		^7^Be	N.D. (<2)	< 5 × 10^−1^	
(5)		31.2		^7^Be	5 ± 5 × 10^−1^	1	
(6)		20.8	389 *	^7^Be	5 ± 5 × 10^−1^	1	
(7)		52 (in specimen)	200	^7^Be	14 ± 5 × 10^−1^	1	
				^3^H	N.D. (<1 × 10^−1^)	< 1 × 10^−2^	
(8)	Polymer gel	10.4	120 *	^7^Be	N.D. (<2)	< 5 × 10^−1^	
(9)		10.4		^7^Be	N.D. (<2)	< 5 × 10^−1^	
(10)	Water	10.4		^7^Be	N.D. (<2)	< 5 × 10^−1^	

* For the calculation method see the footnote of Table 2. ** We tried to measure the activity of ^24^Na but could not detect the corresponding energy spectra in any experiment. Therefore, ^24^Na is not included in this table. *** Radioactivity concentration was calculated based on a conservative estimation by assuming that all radionuclides were located in the irradiated region of the specimen. When the nuclide was not detected (N.D.), the detection limit of the measurement was used instead to calculate the activity concentration.
